# Effect of stress on learning and motivation-relevance to autism spectrum disorder

**DOI:** 10.1177/2058738419856760

**Published:** 2019-06-21

**Authors:** Theoharis C Theoharides, Maria Kavalioti

**Affiliations:** 1 Laboratory of Molecular Immunopharmacology and Drug Discovery, Department of Immunology, Tufts University School of Medicine, Boston, MA, USA; 2 Sackler School of Graduate Biomedical Sciences, Tufts University, Boston, MA, USA; 3 Department of Internal Medicine, Tufts University School of Medicine and Tufts Medical Center, Boston, MA, USA; 4 Biomedical Science Program, University of Greenwich, London, UK; 5 BrainGate, Thessaloniki, Greece

**Keywords:** amygdala, corticotropin-releasing hormone, learning, mast cells, stress

## Abstract

Learning and motivation are critical in the development of children, and to their acquisition of knowledge and skills. A case in point is autism spectrum disorder (ASD), a neurodevelopmental condition characterized by impaired social interactions and communication, as well as by stereotypic movements. Maternal stress has been strongly associated with increased risk of developing ASD. Children experience multiple stressors such as separation anxiety, fear of the unknown, physical and/or emotional trauma, bullying, as well as environmental exposures. Stress is well known to affect learning and motivation. However, patients with ASD have aggrevated tresponses to stress, especially fear response. There is extensive literature connecting the amygdala to social behavior and to pathophysiologic responses to stress. The amygdala regulate the responses to stress, and anatomical changes in amygdala have been reported in ASD. In particular, corticotropin-releasing hormone (CRH), which is secreted under stress, is high in children with ASD and stimulates both mast cells and microglia, thus providing possible targets for therapy. Factors and/or circumstances that could interfere with the neurodevelopmental pathways involved in learning and motivation are clearly important and should be recognized early.

Learning and motivation are critical in the development of children, and to their acquisition of knowledge and skills. Therefore, identifying factors and/or circumstances that could interfere with the neurodevelopmental pathways involved in learning and motivation are clearly important.

All children experience multiple stressors such as (a) separation anxiety, (b) fear of the unknown, (c) difficulty in understanding abstract principles, (d) physical and/or emotional trauma, (e) bullying, (f) punishments, as well as environmental exposures.^1^ Some of these stressors may be more significant than others, especially in children with autism spectrum disorder (ASD). In addition, physiological stressors may be important. For instance, it has been documented that atopic diseases,^2^ such as allergies,^3,4^ asthma,^5^ and eczema,^6^ during childhood are significantly associated with behavioral and learning difficulties, including attention deficit hyperactivity disorder (ADHD) and ASD.

Stress is well known to affect learning and motivation.^7,8^ One study showed that maternal stress during pregnancy due to sudden floods in Australia predicted (at 30-month-old offspring) worse theory of mind, which is an important aspect of child development and successful social functioning.^9^ It is interesting that the key stress hormone, corticotropin-releasing hormone (CRH), was shown to mediate the effect of stress on learning.^10^ CRH is typically secreted from the hypothalamus under stress and activates the hypothalamic–pituitary–adrenal (HPA) axis.^11^ We showed that a unique immune cell, called the mast cell,^12^ can express specific receptors for CRH.^13^ Activation of CRH receptors induced production of vascular endothelial growth factor (VEGF).^14^ Mast cells are juxtaposed to CRH-positive nerve endings in the median eminence of the hypothalamus.^15^ Acute stress and locally secreted CRH stimulates mast cells^16,17^ leading to increased vascular permeability,^18,19^ and disruption of the blood–brain barrier.^20,21^

Anxiety was also strongly correlated with behavior and learning disabilities in children with ASD.^22,23^ ASD patients are prone to stress,^24^ and considerable evidence indicates that patients with ASD have eggagerated responses to threatening images.^25^ Prenatal stress was linked to increased risk of a child developing ASD.^26,27^ ASD is a neurodevelopmental condition characterized by impaired social interactions and communication, as well as by stereotypic movements.^28–31^ ASD affects 1 in 59 children and is projected to reach 1 in 40 children by 2020.^32^ Bauman and Kemper^33^ first identified neuropathologic changes in the amygdala of the postmortem brains of patients with ASD, which has been associated with dysfunctional connectivity in the amygdala.^34,35^ In children with ASD, the amygdala undergo rapid early growth as evidenced by higher spine density than age-matched normotypic controls.^36^ Moreover, children with ASD show an initial excess of neurons in the basal amygdala with a reduction in adulthood, while normal controls have fewer neurons in childhood, but a greater number in adulthood.^37^ These differences in the brain volume and circuitry central to emotional processing may possibly explain the dysregulated “fear response” that many ASD patients exhibit.^33,38^

There is extensive literature from animals and humans connecting the amygdala to social behavior^33,39^ and to pathophysiologic responses to stress.^40^ Infants are well known to instinctively recognize threatening images, an innate fear response programmed in the amygdala.^41,42^ Amygdala are critical for responses to normally fear-inducing stimuli.^43,44^ Stress could affect the amygdala, especially its basolateral (BLA) and medial nuclei, both of which are involved in predator odor-induced fear. Studies in nonhuman primates showed that neonatal amygdala lesions compromise emotional processing.^45,46^

## Conclusion

Given the above, it becomes imperative to address any anxiety-producing environment, as well as reduce the effect of stess, as much as possible.^47^ Teachers and counselors can play an important role, recognizing and minimizing stressors early before any medicinal interventions become necessary. Unfortunately, there are no safe anti-anxiety medications to be used in children. If need be, children with allergies could be prescribed the histamine-1 receptor antagonist hydroxyzine, which also has calming properties or, for children without asthma, the anti-hypertensive medication propranolol, which has strong anti-anxiety actions without clouding mental abilities.^48^ Behavioral modification techniques and some natural products may be useful. For instance, Valeriana or Valeriana/Paciflora exctract could be useful, but it has a short duration (about 2 h) and is sedating. A new dietary supplement combines the anti-oxidant and anti-inflammatory actions of the flavonoid luteolin^49^ together with the anti-anxiety actions of Ashwagandha.^50^
